# Diethyl 2-{[(4-meth­oxy-3-pyrid­yl)amino]­methyl­idene}malonate

**DOI:** 10.1107/S1600536811026353

**Published:** 2011-07-09

**Authors:** Zhi-Fang Zhang

**Affiliations:** a School of Chemistry and Chemical Engineering, Yulin University, Yulin 719000, People’s Republic of China

## Abstract

In the title mol­ecule, C_14_H_8_N_2_O_5_, the amino group is involved in the formation an intra­molecular N—H⋯O hydrogen bond. In the crystal, weak inter­molecular C—H⋯O and C—H⋯N hydrogen bonds link the mol­ecules into ribbons along the *b* axis.

## Related literature

For details of the synthesis, see: Brown & Dewar (1978 ▶). For related structures, see: Thenmozhi *et al.* (2009 ▶); Feng *et al.* (2010 ▶). For potential applications of metal complexes with β-diketone derivatives, see: Nishihama *et al.* (2001 ▶); Soldatov *et al.* (2003 ▶). For bond-length data, see: Allen *et al.* (1987 ▶).
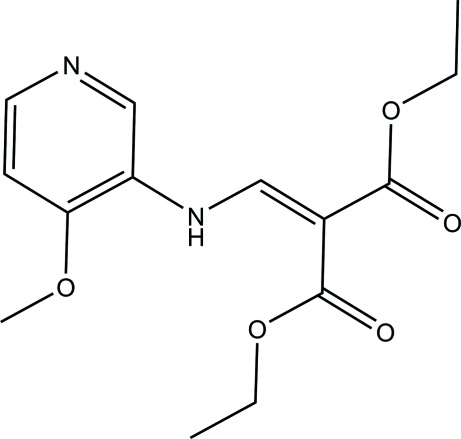


## Experimental

### 

#### Crystal data

C_14_H_18_N_2_O_5_
*M* *_r_* = 294.30
Monoclinic, 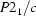

*a* = 19.012 (4) Å
*b* = 8.6620 (17) Å
*c* = 9.1600 (18) Å
β = 94.08 (3)°
*V* = 1504.7 (5) Å^3^
*Z* = 4
Mo *K*α radiation
μ = 0.10 mm^−1^
*T* = 295 K
0.30 × 0.20 × 0.10 mm

#### Data collection

Enraf–Nonius CAD-4 diffractometer
Absorption correction: ψ scan (North *et al.*, 1968 ▶) *T*_min_ = 0.971, *T*_max_ = 0.990
2843 measured reflections
2758 independent reflections
1452 reflections with *I* > 2σ(*I*)
*R*_int_ = 0.034
3 standard reflections every 200 reflections  intensity decay: 1%

#### Refinement

*R*[*F* ^2^ > 2σ(*F* ^2^)] = 0.059
*wR*(*F* ^2^) = 0.142
*S* = 1.01
2758 reflections
190 parameters
H-atom parameters constrained
Δρ_max_ = 0.16 e Å^−3^
Δρ_min_ = −0.14 e Å^−3^

### 

Data collection: *CAD-4 Software* (Enraf–Nonius, 1985 ▶); cell refinement: *CAD-4 Software*; data reduction: *XCAD4* (Harms & Wocadlo, 1995 ▶); program(s) used to solve structure: *SHELXS97* (Sheldrick, 2008 ▶); program(s) used to refine structure: *SHELXL97* (Sheldrick, 2008 ▶); molecular graphics: *SHELXTL* (Sheldrick, 2008 ▶); software used to prepare material for publication: *SHELXTL*.

## Supplementary Material

Crystal structure: contains datablock(s) I, global. DOI: 10.1107/S1600536811026353/cv5129sup1.cif

Structure factors: contains datablock(s) I. DOI: 10.1107/S1600536811026353/cv5129Isup2.hkl

Supplementary material file. DOI: 10.1107/S1600536811026353/cv5129Isup3.cml

Additional supplementary materials:  crystallographic information; 3D view; checkCIF report

## Figures and Tables

**Table 1:** Hydrogen-bond geometry (Å, °)

*D*—H⋯*A*	*D*—H	H⋯*A*	*D*⋯*A*	*D*—H⋯*A*
N2—H2*A*⋯O4	0.86	2.01	2.656 (3)	131
C6—H6*A*⋯O2^i^	0.96	2.57	3.462 (4)	155
C2—H2*B*⋯N1^ii^	0.93	2.63	3.389 (3)	139

Symmetry codes: (i) 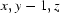; (ii) 
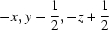
.

**Fig. 1.**
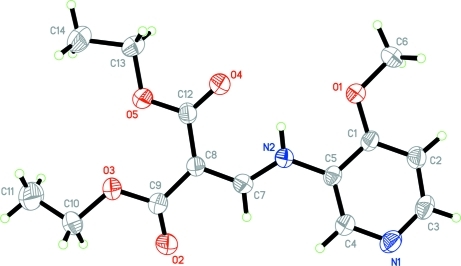
The molecular structure of (I) showing the atomic numbering and 30% probabilty displacement ellipsoids.

## supplementary crystallographic information

### Comment

β-Diketone as an chelating group has been widely used in supramolecular chemistry. Their metal complexes are used for the separation of elements with similar properties (Nishihama *et al.*, 2001). These metal complexes have applications in materials science or act as NMR shift reagents (Soldatov *et al.*, 2003). The tittle compound, (I), is a derivative of β-diketone. Herewith we present its crystal structure.

In (I) (Fig. 1), the amino group is involved in formation an intramolecular N—H···O hydrogen bond (Table 1). The bond lengths and angles are within normal ranges (Allen *et al.*, 1987) and correspond to those observed in the related compounds (Thenmozhi *et al.*, 2009; Feng *et al.*, 2010).

In the crystal structure, weak intermolecular C—H···O and C—H···N hydrogen bonds (Table 1) link molecules into approximately planar ribbons along the *b* axis.

### Experimental

The title compound was synthesized according to the method proposed by Brown & Dewar (1978). A mixture of 3-Nitro-4-methoxypyridine (5 g; 0.0325 mol), 10% palladium on carbon (500 mg), and dry methanol (125 ml) was hydrogenated for 6 h in a Parr apparatus at 50 psi. Filtration of the mixture through Celite and evaporation of the filtrate yielded the crude amine as a light tan oil or solid. The amine was stirred and refluxed in toluene (100 ml) with ethoxymethylenemalonic ester(EMME; 7 g; 0.0325 mol) for 24 hand then the reaction mixture was evaporated to dryness. The residue was dissolved in boiling Skelly B, filtered by gravity, and cooled to room temperature. The title compound crystallized as fine, white platelets.

### Refinement

H atoms were positioned geometrically [N—H 0.86 Å; C—H 0.93-0.97 Å], and constrained to ride on their parent atoms, with *U*_iso_(H) = 1.2-1.5 *U*_eq_(C, N).

### Crystal data

**Table:**

C_14_H_18_N_2_O_5_	*F*(000) = 624
*M**_r_* = 294.30	*D*_x_ = 1.299 Mg m^−^^3^
Monoclinic, *P*2_1_/*c*	Mo *K*α radiation, λ = 0.71073 Å
Hall symbol: -P 2ybc	Cell parameters from 25 reflections
*a* = 19.012 (4) Å	θ = 10–13°
*b* = 8.6620 (17) Å	µ = 0.10 mm^−^^1^
*c* = 9.1600 (18) Å	*T* = 295 K
β = 94.08 (3)°	Block, colourless
*V* = 1504.7 (5) Å^3^	0.30 × 0.20 × 0.10 mm
*Z* = 4	

### Data collection

**Table:**

Enraf–Nonius CAD-4 diffractometer	1452 reflections with *I* > 2σ(*I*)
Radiation source: fine-focus sealed tube	*R*_int_ = 0.034
graphite	θ_max_ = 25.4°, θ_min_ = 1.1°
ω/2θ scans	*h* = −22→0
Absorption correction: ψ scan (North *et al.*, 1968)	*k* = 0→10
*T*_min_ = 0.971, *T*_max_ = 0.990	*l* = −10→11
2843 measured reflections	3 standard reflections every 200 reflections
2758 independent reflections	intensity decay: 1%

### Refinement

**Table:**

Refinement on *F*^2^	Primary atom site location: structure-invariant direct methods
Least-squares matrix: full	Secondary atom site location: difference Fourier map
*R*[*F*^2^ > 2σ(*F*^2^)] = 0.059	Hydrogen site location: inferred from neighbouring sites
*wR*(*F*^2^) = 0.142	H-atom parameters constrained
*S* = 1.01	*w* = 1/[σ^2^(*F*_o_^2^) + (0.056*P*)^2^] where *P* = (*F*_o_^2^ + 2*F*_c_^2^)/3
2758 reflections	(Δ/σ)_max_ < 0.001
190 parameters	Δρ_max_ = 0.16 e Å^−^^3^
0 restraints	Δρ_min_ = −0.14 e Å^−^^3^

### Special details

**Table:**

Geometry. All e.s.d.'s (except the e.s.d. in the dihedral angle between two l.s. planes) are estimated using the full covariance matrix. The cell e.s.d.'s are taken into account individually in the estimation of e.s.d.'s in distances, angles and torsion angles; correlations between e.s.d.'s in cell parameters are only used when they are defined by crystal symmetry. An approximate (isotropic) treatment of cell e.s.d.'s is used for estimating e.s.d.'s involving l.s. planes.
Refinement. Refinement of *F*^2^ against ALL reflections. The weighted *R*-factor *wR* and goodness of fit *S* are based on *F*^2^, conventional *R*-factors *R* are based on *F*, with *F* set to zero for negative *F*^2^. The threshold expression of *F*^2^ > σ(*F*^2^) is used only for calculating *R*-factors(gt) *etc*. and is not relevant to the choice of reflections for refinement. *R*-factors based on *F*^2^ are statistically about twice as large as those based on *F*, and *R*- factors based on ALL data will be even larger.

### Fractional atomic coordinates and isotropic or equivalent isotropic displacement parameters (Å^2^)

**Table:**

	*x*	*y*	*z*	*U*_iso_*/*U*_eq_	
O1	0.15081 (10)	0.3780 (2)	0.5511 (2)	0.0649 (6)	
C1	0.11221 (14)	0.4906 (3)	0.4828 (3)	0.0502 (7)	
N1	0.03465 (13)	0.7408 (3)	0.3671 (3)	0.0668 (7)	
N2	0.17585 (11)	0.6573 (2)	0.6532 (2)	0.0527 (6)	
H2A	0.1916	0.5742	0.6954	0.063*	
O2	0.25551 (12)	1.0765 (2)	0.7731 (2)	0.0826 (8)	
C2	0.06290 (15)	0.4714 (3)	0.3677 (3)	0.0591 (8)	
H2B	0.0543	0.3744	0.3268	0.071*	
O3	0.31389 (12)	0.9837 (2)	0.9718 (2)	0.0868 (8)	
C3	0.02656 (15)	0.5984 (4)	0.3142 (3)	0.0645 (9)	
H3A	−0.0062	0.5840	0.2350	0.077*	
O4	0.26443 (12)	0.5409 (2)	0.8621 (2)	0.0804 (7)	
C4	0.08378 (14)	0.7576 (3)	0.4773 (3)	0.0590 (8)	
H4A	0.0916	0.8563	0.5150	0.071*	
O5	0.34811 (10)	0.6944 (2)	0.9616 (2)	0.0662 (6)	
C5	0.12345 (14)	0.6392 (3)	0.5386 (3)	0.0463 (7)	
C6	0.14403 (17)	0.2247 (3)	0.4922 (4)	0.0776 (10)	
H6A	0.1740	0.1558	0.5503	0.116*	
H6B	0.1576	0.2245	0.3933	0.116*	
H6C	0.0959	0.1913	0.4937	0.116*	
C7	0.20362 (14)	0.7889 (3)	0.7033 (3)	0.0528 (7)	
H7A	0.1854	0.8789	0.6601	0.063*	
C8	0.25623 (14)	0.8065 (3)	0.8119 (3)	0.0484 (7)	
C9	0.27475 (15)	0.9670 (3)	0.8476 (3)	0.0591 (8)	
C10	0.3349 (2)	1.1375 (4)	1.0158 (5)	0.1076 (14)	
H10A	0.3393	1.2015	0.9301	0.129*	
H10B	0.2996	1.1831	1.0739	0.129*	
C11	0.4004 (2)	1.1295 (4)	1.0990 (5)	0.1279 (16)	
H11A	0.4144	1.2313	1.1305	0.192*	
H11B	0.4353	1.0869	1.0399	0.192*	
H11C	0.3958	1.0649	1.1829	0.192*	
C12	0.28810 (15)	0.6696 (3)	0.8807 (3)	0.0526 (7)	
C13	0.38151 (16)	0.5613 (3)	1.0303 (3)	0.0721 (9)	
H13A	0.4013	0.4961	0.9576	0.087*	
H13B	0.3475	0.5014	1.0804	0.087*	
C14	0.43854 (18)	0.6193 (4)	1.1373 (4)	0.0959 (12)	
H14A	0.4621	0.5333	1.1855	0.144*	
H14B	0.4183	0.6836	1.2086	0.144*	
H14C	0.4719	0.6781	1.0864	0.144*	

### Atomic displacement parameters (Å^2^)

**Table:**

	*U*^11^	*U*^22^	*U*^33^	*U*^12^	*U*^13^	*U*^23^
O1	0.0708 (14)	0.0499 (12)	0.0701 (13)	0.0029 (10)	−0.0218 (11)	−0.0015 (10)
C1	0.0463 (16)	0.0575 (17)	0.0455 (15)	0.0011 (14)	−0.0067 (13)	0.0004 (13)
N1	0.0626 (17)	0.0728 (17)	0.0629 (16)	0.0032 (14)	−0.0106 (13)	0.0104 (14)
N2	0.0607 (15)	0.0465 (14)	0.0490 (13)	0.0003 (12)	−0.0088 (11)	−0.0020 (11)
O2	0.1070 (19)	0.0537 (13)	0.0823 (16)	0.0006 (12)	−0.0257 (14)	0.0033 (11)
C2	0.0626 (19)	0.0608 (19)	0.0523 (17)	−0.0071 (16)	−0.0065 (15)	−0.0006 (15)
O3	0.1061 (18)	0.0546 (13)	0.0922 (17)	−0.0018 (12)	−0.0465 (14)	−0.0092 (11)
C3	0.060 (2)	0.080 (2)	0.0512 (18)	−0.0070 (17)	−0.0126 (15)	0.0049 (16)
O4	0.0954 (17)	0.0519 (13)	0.0881 (16)	−0.0083 (12)	−0.0342 (13)	0.0069 (11)
C4	0.0562 (19)	0.0590 (18)	0.0601 (18)	−0.0002 (15)	−0.0084 (15)	0.0010 (15)
O5	0.0625 (13)	0.0543 (12)	0.0786 (14)	−0.0005 (10)	−0.0184 (11)	0.0023 (10)
C5	0.0471 (16)	0.0533 (17)	0.0379 (14)	−0.0012 (14)	−0.0017 (12)	0.0030 (12)
C6	0.086 (2)	0.0476 (19)	0.096 (3)	0.0027 (17)	−0.0172 (19)	−0.0094 (17)
C7	0.0563 (18)	0.0485 (17)	0.0533 (17)	0.0021 (15)	0.0018 (14)	−0.0020 (14)
C8	0.0485 (16)	0.0480 (16)	0.0482 (15)	−0.0025 (14)	0.0003 (13)	−0.0016 (13)
C9	0.0544 (19)	0.059 (2)	0.0629 (19)	−0.0010 (16)	−0.0062 (15)	−0.0029 (16)
C10	0.115 (3)	0.055 (2)	0.142 (3)	−0.004 (2)	−0.063 (3)	−0.021 (2)
C11	0.120 (3)	0.090 (3)	0.164 (4)	−0.022 (3)	−0.063 (3)	−0.002 (3)
C12	0.0569 (19)	0.0520 (18)	0.0481 (16)	−0.0066 (15)	−0.0020 (15)	−0.0035 (14)
C13	0.071 (2)	0.069 (2)	0.073 (2)	0.0150 (18)	−0.0134 (18)	0.0041 (17)
C14	0.078 (2)	0.110 (3)	0.095 (3)	0.014 (2)	−0.028 (2)	0.000 (2)

### Geometric parameters (Å, °)

**Table:**

O1—C1	1.348 (3)	C6—H6A	0.9600
O1—C6	1.436 (3)	C6—H6B	0.9600
C1—C2	1.370 (3)	C6—H6C	0.9600
C1—C5	1.396 (3)	C7—C8	1.368 (3)
N1—C3	1.330 (4)	C7—H7A	0.9300
N1—C4	1.333 (3)	C8—C12	1.455 (4)
N2—C7	1.325 (3)	C8—C9	1.465 (4)
N2—C5	1.404 (3)	C10—C11	1.414 (4)
N2—H2A	0.8600	C10—H10A	0.9700
O2—C9	1.210 (3)	C10—H10B	0.9700
C2—C3	1.371 (4)	C11—H11A	0.9600
C2—H2B	0.9300	C11—H11B	0.9600
O3—C9	1.323 (3)	C11—H11C	0.9600
O3—C10	1.440 (3)	C13—C14	1.495 (4)
C3—H3A	0.9300	C13—H13A	0.9700
O4—C12	1.209 (3)	C13—H13B	0.9700
C4—C5	1.370 (3)	C14—H14A	0.9600
C4—H4A	0.9300	C14—H14B	0.9600
O5—C12	1.333 (3)	C14—H14C	0.9600
O5—C13	1.440 (3)		
			
C1—O1—C6	117.6 (2)	C7—C8—C9	114.8 (2)
O1—C1—C2	126.1 (3)	C12—C8—C9	126.2 (2)
O1—C1—C5	115.6 (2)	O2—C9—O3	121.9 (3)
C2—C1—C5	118.2 (3)	O2—C9—C8	124.0 (3)
C3—N1—C4	115.7 (3)	O3—C9—C8	114.1 (3)
C7—N2—C5	126.8 (2)	C11—C10—O3	108.8 (3)
C7—N2—H2A	116.6	C11—C10—H10A	109.9
C5—N2—H2A	116.6	O3—C10—H10A	109.9
C1—C2—C3	118.5 (3)	C11—C10—H10B	109.9
C1—C2—H2B	120.7	O3—C10—H10B	109.9
C3—C2—H2B	120.7	H10A—C10—H10B	108.3
C9—O3—C10	118.0 (2)	C10—C11—H11A	109.5
N1—C3—C2	124.9 (3)	C10—C11—H11B	109.5
N1—C3—H3A	117.6	H11A—C11—H11B	109.5
C2—C3—H3A	117.6	C10—C11—H11C	109.5
N1—C4—C5	124.4 (3)	H11A—C11—H11C	109.5
N1—C4—H4A	117.8	H11B—C11—H11C	109.5
C5—C4—H4A	117.8	O4—C12—O5	121.5 (3)
C12—O5—C13	116.6 (2)	O4—C12—C8	123.4 (3)
C4—C5—C1	118.3 (2)	O5—C12—C8	115.0 (2)
C4—C5—N2	124.3 (2)	O5—C13—C14	107.1 (3)
C1—C5—N2	117.3 (2)	O5—C13—H13A	110.3
O1—C6—H6A	109.5	C14—C13—H13A	110.3
O1—C6—H6B	109.5	O5—C13—H13B	110.3
H6A—C6—H6B	109.5	C14—C13—H13B	110.3
O1—C6—H6C	109.5	H13A—C13—H13B	108.5
H6A—C6—H6C	109.5	C13—C14—H14A	109.5
H6B—C6—H6C	109.5	C13—C14—H14B	109.5
N2—C7—C8	126.9 (3)	H14A—C14—H14B	109.5
N2—C7—H7A	116.5	C13—C14—H14C	109.5
C8—C7—H7A	116.5	H14A—C14—H14C	109.5
C7—C8—C12	119.0 (2)	H14B—C14—H14C	109.5
			
C6—O1—C1—C2	5.0 (4)	N2—C7—C8—C12	1.7 (4)
C6—O1—C1—C5	−176.5 (2)	N2—C7—C8—C9	−178.1 (3)
O1—C1—C2—C3	177.8 (3)	C10—O3—C9—O2	−1.4 (5)
C5—C1—C2—C3	−0.7 (4)	C10—O3—C9—C8	179.8 (3)
C4—N1—C3—C2	2.2 (4)	C7—C8—C9—O2	−12.7 (4)
C1—C2—C3—N1	−1.1 (5)	C12—C8—C9—O2	167.5 (3)
C3—N1—C4—C5	−1.7 (4)	C7—C8—C9—O3	166.0 (3)
N1—C4—C5—C1	0.1 (4)	C12—C8—C9—O3	−13.8 (4)
N1—C4—C5—N2	179.1 (3)	C9—O3—C10—C11	−150.4 (4)
O1—C1—C5—C4	−177.5 (2)	C13—O5—C12—O4	−2.5 (4)
C2—C1—C5—C4	1.1 (4)	C13—O5—C12—C8	−179.5 (2)
O1—C1—C5—N2	3.4 (4)	C7—C8—C12—O4	−10.7 (4)
C2—C1—C5—N2	−177.9 (2)	C9—C8—C12—O4	169.1 (3)
C7—N2—C5—C4	−12.2 (4)	C7—C8—C12—O5	166.2 (2)
C7—N2—C5—C1	166.8 (3)	C9—C8—C12—O5	−14.0 (4)
C5—N2—C7—C8	−177.8 (3)	C12—O5—C13—C14	−168.9 (3)

### Hydrogen-bond geometry (Å, °)

**Table:**

*D*—H···*A*	*D*—H	H···*A*	*D*···*A*	*D*—H···*A*
N2—H2A···O4	0.86	2.01	2.656 (3)	131
C6—H6A···O2^i^	0.96	2.57	3.462 (4)	155
C2—H2B···N1^ii^	0.93	2.63	3.389 (3)	139

Symmetry codes: (i) *x*, *y*−1, *z*; (ii) −*x*, *y*−1/2, −*z*+1/2.
